# Acid Stable Yeast Cell-Associated Tannase with High Capability in Gallated Catechin Biotransformation

**DOI:** 10.3390/microorganisms9071418

**Published:** 2021-06-30

**Authors:** Nalapat Leangnim, Jakkrit Aisara, Kridsada Unban, Chartchai Khanongnuch, Apinun Kanpiengjai

**Affiliations:** 1 Graduate Program in Biotechnology, The Graduate School, Chiang Mai University, Chiang Mai 50200, Thailand; nalapat_l@cmu.ac.th (N.L.); jakkrit_ai@cmu.ac.th (J.A.); 2 Department of Chemistry, Division of Biochemistry and Biochemical Technology, Faculty of Science, Chiang Mai University, Chiang Mai 50200, Thailand; 3 Division of Biotechnology, School of Agro-Industry, Faculty of Agro-Industry, Chiang Mai University, Chiang Mai 50100, Thailand; kridsada_u@cmu.ac.th (K.U.); chartchai.k@cmu.ac.th (C.K.); 4 Research Center for Multidisciplinary Approaches to Miang, Chiang Mai University, Chiang Mai 50200, Thailand

**Keywords:** cell-associated tannase, tannase, yeast, tannins, catechin, miang, tea

## Abstract

Previously, nine tannin-tolerant and tannase-producing yeasts were isolated from Miang; all produced cell-associated tannase (CAT) during growth in tannin substrate. Among which, only CAT from *Sporidiobolus ruineniae* showed better stability than its purified form. Yet, it is of particular interest to directly characterize CATs from the latter yeasts. In this study, four CATs from yeasts, namely *Cyberlindnera rhodanensis* A22.3, *Candida* sp. A39.3, *Debaryomyces hansenii* A45.1, and *Cy. rhodanensis* A45.3 were characterized. The results indicate that all CATs were produced within the same production yield (11 mU/mL). Most CATs exhibited similar pH and temperature optima and stabilities, except for CAT from *Cy. rhodanensis* A22.3. This CAT was assigned as acid-stable tannase due to its unusual optimum pH of 2.0 with pH stability and half-life thermostability in the range of pH 2.0–4.0, and 70 °C, respectively. All CATs demonstrated high substrate specificity toward epigallocatechin gallate and epicatechin gallate, thus forming epigallocatechin and epicatechin, respectively. Moreover, they showed operational stability to repeated use for up to five cycles without loss of the initial activity. Therefore, CATs from these yeasts could be useful for the extraction and biotransformation of tea catechins and related applications.

## 1. Introduction

Tannins are naturally found in plant materials as water-soluble polyphenols with varying molecular weight [1]. They have been classified into three categories including hydrolysable tannins, condensed tannins, and complex tannins. The latter ones are an intermediate group that share a structure combination between hydrolysable tannins and condensed tannins. To form complex tannins, catechin or epicatechin units are linked via glycosidic linkage to a gallotannin or an ellagitannin unit, thus commonly referred to as catechin tannins, the form that is generally found in tea leaves and tropical shrub legumes [2]. Either condensed or complex tannins are considered microbial inhibitors as they resist microbial attacks and are toxic to a variety of microorganisms [3]. In addition, catechin tannins are responsible for the astringent and bitter tastes of tea leaves [4].

Tannin acyl hydrolase (EC 3.1.1.20) is commonly known as tannase, an enzyme that catalyzes the hydrolysis of ester bonds present in gallotannin, complex tannins, and gallate esters to release gallic acid. To date, fungi are the major source of microbial tannase, particularly *Aspergillus* sp. and *Penicillium* sp. Bacteria are the second major source of tannase [5], whereas only a few yeasts have been reported to produce tannase, including *Candida* sp. [6], *Aureobasidium melanogenum* [7], *Blastobotrys* (*Arxula*) *adeninivorans* [8], *Kluyveromyces marxianus* [9], *Sporidiobolus ruineniae* [10], and *Rhodosporidium diobovatum* [11]. Tannases are widely used in various industrial applications including gallic acid production, instant tea production, beer and juice clarification, and effluent treatment [2]. Not only free tannases were industrially applied, but also immobilized enzymes were trialed to reduce some processes and operating costs associated with the production. Immobilized enzymes provide a number of advantages such as an easy separation of the enzymes, a reduction in the cost of downstream processing, the multiple use of the enzymes (recycling), and a better stability toward hazardous conditions [12].

In our previous research study, nine yeast strains isolated from Miang indicated as tannin-tolerant and tannase-producing yeasts belonging to four different species, namely *Cyberlindnera rhodanensis* (strains A22.3, and A45.3), *Debaryomyces hansenii* (strains A28.1, A28.2, A42, A45.1 and A46), *Candida* sp. A39.3, and *Sporidiobolus ruineniae* A45.2 [13]. They have been confirmed to produce tannase, which is translocated and immobilized onto their cell wall. Yet, these tannases are classified as cell-associated tannases (CATs). A previous study revealed that CAT of *S. ruineniae* A45.2 is active and more stable toward pH and temperature changes when it occurs on the cell form compared to the free form. In addition, its steady-state kinetic constants, particularly *K*_m_, were not significantly different to those of the purified enzyme [10].

Miang is a fermented food product prepared from the tea leaves of *Camellia sinensis* var. *assamica* and is traditionally produced in the mountainous areas of northern Thailand. It has a long history that is indicative of a sociocultural relationship with northern Thai people. Unlike other types of tea, Miang is a unique product that is known as chewing tea or eating tea, but it has become less popular among younger generations [14]. The ethnic roots of the Miang production process in northern Thailand is a potential source of a number of health-related bioactive compounds [15,16,17], specifically tea catechins which continue to attract research interest. However, the Miang production process must be conserved and/or develop to produce a more acceptable product for younger generations. 

The refinement of Miang tea products can be considered a way of conserving the local wisdom of northern Thai people. To make the product unique and different from other tea products, the release of the bioactive compounds in Miang tea infusion should be further developed and promoted. Tannase has been purposed as the potential key in liberating various bioactive compounds, particularly epigallocatechin gallate (EGCG) and epicatechin gallate (ECG). Therefore, the research challenge of this work is to develop an acceptable Miang tea product that is similar in popularity to Taiwanese tea and Japanese green tea. It is expected that the developed Miang tea would be more acceptable by younger generations while providing various health benefits.

In this context, we characterized the enzyme properties of the remaining CATs from four representative yeast species including *Cy*. *rhodanensis* A22.3, *Cy*. *rhodanensis* A45.3, *D*. *hansenii* A45.1, and *Candida* sp. A39.3. Additionally, we demonstrated their applicable features.

## 2. Materials and Methods

### 2.1. Chemicals and Culture Media

High-performance liquid chromatography (HPLC) grade EGCG, ECG, epigallocatechin (EGC), epicatechin (EC), and gallic acid (GA) were purchased from Sigma-Aldrich (St. Louis, MO, USA). Tannic acid, methyl gallate, and propyl gallate were of analytical grade and purchased from Sigma-Aldrich. All chemicals used for buffer preparation, cations, and organic solvents were of analytical grade and obtained from RCI Labscan (Bangkok, Thailand). The medium ingredients used in this study, such as agar, yeast extract, peptone, and malt extract, were purchased from HiMedia (Nashik, India).

### 2.2. Microorganisms and Culture Conditions

The microorganisms used were obtained from our previous studies [13] and included *Cyberlindnera rhodanensis* A22.3, *Candida* sp. A39.3, *Debaryomyces hansenii* A45.1, and *Cy. rhodanensis* A45.3. All yeast strains were grown on yeast-malt agar (YMA) (3 g/L yeast extract, 3 g/L malt extract, 10 g/L glucose, 30 g/L agar) supplemented with 1.0% (w/v) filter sterile tannic acid at 30 °C for 24–48 h. For inoculum preparation, a single colony of each yeast strain was spiked in yeast-malt broth (YMB) and incubated at 30 °C on a 150-rpm rotary shaker for 24 h or until the culture reached a maximal optical density at 600 nm (OD_600_) of 8–9. A 10% (v/v) inoculum size was used for cultivation in all experiments.

### 2.3. Effect of Temperature on Growth of Tannin-Tolerant Yeasts

Four strains of yeasts were grown in YMB at 30, 37, 39, 42, and 45 °C on a 150-rpm rotary shaker. After 24 h of cultivation, for each culture, turbidity was measured at 600 nm.

### 2.4. Production of CAT from Tannin-Tolerant Yeasts

Seed inoculum of 10% (v/v) was transferred to a 250-mL Erlenmeyer flask containing 50 mL YMB supplemented with 5 g/L tannic acid. The incubation was carried out on a 150-rpm rotary shaker at 30 °C for 24 h. Samples were taken in intervals to determine viable cells by the spread plate technique, released gallic acid, and CAT activity [10].

### 2.5. CAT Preparation

To prepare cells for CAT activity assay, the culture was centrifuged at 12,000 rpm at 4 °C for 5 min. The supernatant was collected for analysis of gallic acid. Accordingly, the cell pellet was collected and washed twice with 20 mM sodium phosphate buffer pH 6.5 supplemented with 0.1% (v/v) triton X-100 in order to remove any gallic acid attached to the yeast cell wall. Subsequently, the obtained cells were resuspended in 100 mM sodium phosphate buffer pH 6.5 prior to being used as CAT. Here, triton X-100 played an important role in the removal of gallic acid that had been generated into the culture medium. This was likely caused by the enzymatic degradation of tannic acid. A part of the generated gallic acid was then attached to the yeast cell wall, thus interfering with enzyme activity assay and biochemical characterization.

### 2.6. Assay of CAT Activity and Determination of Gallic Acid

The CAT activity was determined according to a previous report [10], with a slight modification. Briefly, 50 μL of yeast cell suspension was mixed with 50 μL substrate (12.5 mM methyl gallate in 100 mM sodium phosphate buffer pH 6.5). The reaction was carried out at 37 °C for 20 min. Then, 60 μL of 0.667% (w/v) methanolic rhodanine was added into the reaction mixture to stop the reaction and to detect the release of gallic acid from tannic acid. After 5 min of incubation at room temperature (25 °C), the pinkish purple color was visualized by adding 40 μL of 0.5 M KOH, and the mixture was left at room temperature for 5 min. Finally, 800 μL of distilled water was added. The mixture was vigorously mixed and centrifuged at 12,000 rpm for 2 min to remove the cell pellet. The clear supernatant was then measured at an absorbance of 520 nm. One unit of CAT was defined as the release of 1 μmol of gallic acid in 1 min under the assay conditions. For characterization, CAT activity was initially set up to approximately 36 mU/mL prior to conducting the experiments.

### 2.7. Effect of pH on CAT Activity and Stability

To investigate the optimal pH of CATs, the enzyme was assayed under the standard assay conditions, but the pH values varied from 1.0 to 9.0, depending on various buffers at 100 mM. Potassium hydroxide-hydrochloric acid was used for pH values ranging from 1.0–2.5, citrate-phosphate buffer was used for pH values between 3.0 and 6.0, and sodium phosphate buffer and Tris-hydrochloric acid were used for pH values ranging from 6.0–8.0 and 8.0–9.0, respectively. The pH value that gave the maximal CAT activity was set as 100% relative activity. For the determination of pH stability, CAT was incubated at various pH values ranging from 1.0 to 9.0 (at 20 mM) at 37 °C for 6 h. The pH values were dependent upon the buffer systems as previously described. After the incubation process, residual CAT activity was determined according to the standard assay conditions. The percentage of residual activity was calculated by setting the initial CAT activity to 100% residual activity.

### 2.8. Effect of Temperature on CAT Activity Stability

To investigate the optimal temperature of CAT, the enzyme was assayed using the standard conditions, but at incubation temperatures of 4, 20, 30, 37, 40, 45, 50, 55, and 60 °C. The temperature that gave the maximal CAT activity was considered as 100% relative activity. For the determination of thermostability, CAT was incubated at temperatures ranging from 4–90 °C for 1 h. After that, it was placed in an ice bath for 5 min prior to determining residual activity according to the standard assay conditions. The percentage residual activity was calculated by setting the initial CAT activity to 100% residual activity.

### 2.9. Effect of Cations on CAT Activity

The CAT was assayed in the presence of different mono-, di-, and trivalent cations, including Na^+^, K^+^, Ba^2+^, Ca^2+^, Mg^2+^, Cu^2+^, Mn^2+^, Co^2+^, Ni^2+^, Zn^2+^, Al^3+^, and Fe^3+^. Each cation was set to a final concentration of 5 mM in the reaction mixture. The CAT activity assayed without cation supplementation was set as 100% relative activity.

### 2.10. Effect of Organic Solvents on CAT Stability

The obtained yeast cell pellet as CAT was resuspended with 20% (v/v) of different organic solvents at the final concentration and incubated at 37 °C. After incubation for 30 min, the suspension was centrifuged at 12,000 rpm at 4 °C for 5 min in order to remove any organic solvents. The cell pellet was resuspended with 100 mM sodium phosphate buffer pH 6.5. This was done with the same volume of the organic solvent that was applied prior to determining the residual CAT activity using standard assay conditions. The percentage residual activity was calculated by setting the initial CAT activity to 100% residual activity.

### 2.11. Substrate Specificity

The CAT activity was assayed with 12.5 mM of different substrates, including methyl gallate (MG), propyl gallate (PG), ECGC, and ECG, under the standard assay conditions. The CAT activity was set as 100% relative activity when assayed with methyl gallate.

### 2.12. Feasible Evaluation for the Biotransformation of Catechin Derivatives

Only EGCG and ECG were evaluated for their feasibility to form other catechin derivatives. Reaction mixtures between each CAT (36 mU/mL) and 12.5 mM EGCG or ECG were conducted; incubation was carried out at 37 °C for 60 min. After that, each reaction mixture was centrifuged at 12,000 rpm, 4 °C for 5 min to remove the cell pellet. The supernatant was filtered through a 0.22-μm filter cartridge prior to direct injection into a high-performance liquid chromatograph. The VertiSepTM UPS C18 column with a dimension of 5 μm × 4.6 mm ID × 150 mm (Vertical Chromatography Co., Ltd., Bangkok, Thailand) was used for the separation of catechin derivatives; it was equilibrated with a mobile phase consisting of a mixture of 0.05% phosphoric acid pH 2.4 (phase A) and a mixture of methanol and acetonitrile (3:2 v/v) (phase B). The linear gradient dynamics were as follows: phase at a ratio of 10% solution B and 90% solution A. The separation was achieved with a linear gradient program as follows: 25% phase B and 75% phase A for 15 min, then increase to 60% phase B and 40% solution A for 25 min. Flow rate and separation temperature were set up at 1 mL/min, 30 °C, respectively. The catechin derivatives were detected by absorbance at 280 nm.

### 2.13. Operational Stability Determination

The operational stability of CATs was determined using the standard assay conditions (pH 6.5, 37 °C), with an incubation time of 20 min in a repeated batch process. Biomass equivalent to 36 U/mL of each CAT was incubated with 12.5 mM methyl gallate. After each operation, CAT was centrifuged at 12,000 rpm, 4 °C, for 5 min and washed with 20 mM citrate-phosphate buffer pH 7.0. The resulting CAT was then subsequently used for another batch operation. Gallic acid production was determined for each batch. The initial concentration of gallic acid obtained from the first batch was set to 100%.

### 2.14. Statistical Analysis

Data analysis of the mean values was performed based on a full-factorial complete randomized design (CRD). Briefly, data were subjected to analysis of variance (ANOVA), and multiple comparison tests were performed based on all paired comparisons, using Tukey’s HSD test at the 95% confidence level. All analyses were carried out using the Statistix software version 8.0 (Analytical Software, Tallahassee, FL, USA). A probability value of *p* < 0.05 was considered significant.

## 3. Results

### 3.1. Effect of Temperature on the Growth of Tannin-Tolerant Yeasts

**Figure 1:**
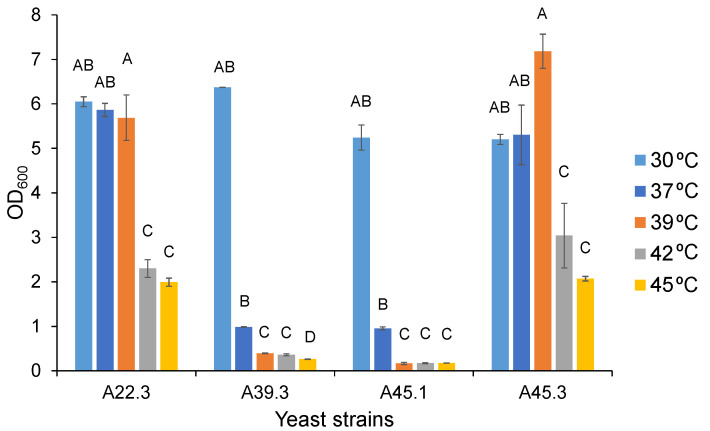
Growth of four CAT-producing yeast strains after being cultivated in YMB at 30, 37, 39, 42, and 45 °C on a 150-rpm rotary shaker for 24 h. Different uppercase letters in columns with the same dept indicate significant differences in OD_600_ values at *p* < 0.05. The resulting values represent the mean values of two independent experiments.

Two strains of *Cy. rhodanensis* (A22.3 and A45.3) had the same range of growth temperature from 30–45 °C, with an optimal temperature from 30–39 °C. Higher temperatures led to significantly low growth. These results contrasted with those found for *Candida* sp. A39.3 and *D. hansenii* A45.1, which had a growth temperature of 30 °C (Figure 1).

### 3.2. Profile of CAT Production by Tannin-Tolerant Yeasts

**Figure 2:**
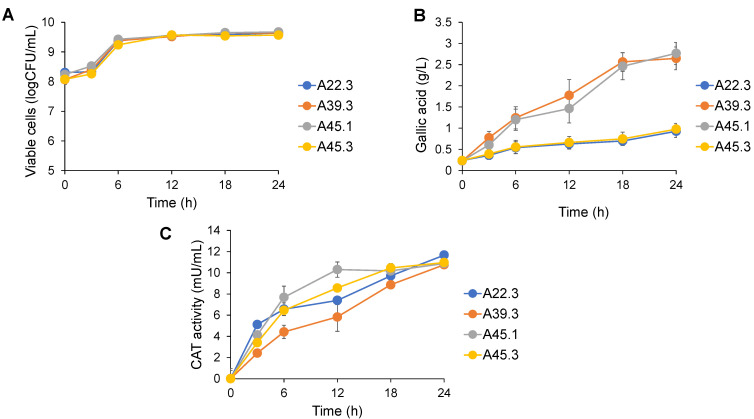
Time course of fermentation for the production of viable cells (**A**), gallic acid (**B**), and CAT (**C**). The conditions were carried in YMB supplemented with 5 g/L tannic acid at 30 °C on a 150-rpm rotary shaker for 24 h. The resulting values represent the mean values of two independent experiments.

All yeasts showed a similar growth pattern and increased cell viability, from 10^9^ to 10^10^ cells/mL. They had a short lag phase and exponential phase (Figure 2A). The gallic acid content released into the culture media ranged from 1.0–3.0 g/L, depending on the yeast strain (Figure 2B). Two strains of *Cy. rhodanensis* produced high amounts of gallic acid, with a similar production pattern. The gallic acid level was dramatically increased up to approximately 2.4 g/L within 18 h and gradually increased up to 24 h of the cultivation. *Candida* sp. A39.3 and *D. hansenii* A45.1 gradually produced gallic acid, with maximum values of 2.6 ± 0.3 and 2.7 ± 0.3 g/L gallic acid, respectively. The tannases produced by the four yeast strains were classified as the cell-associated tannase (CAT). The enzyme production profiles slightly differed among the strains, and the overall CAT activities reached the maximum of approximately 11 mU/mL at 24 h of cultivation (Figure 2C).

### 3.3. Temperature Optima and Stabilities, and pH Optima and Stabilities of CATs

**Figure 3:**
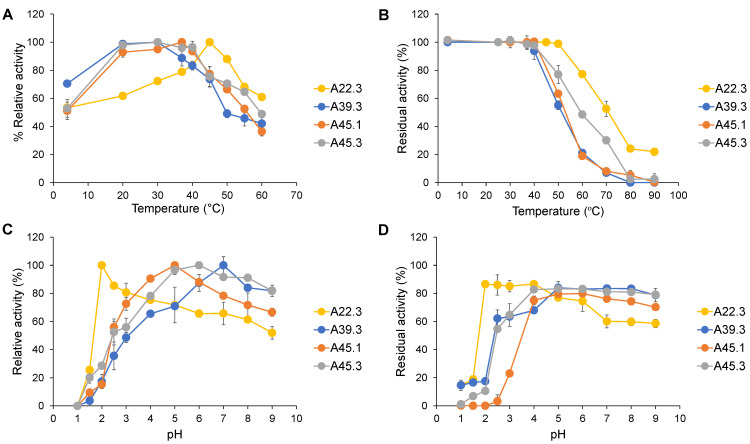
Effect of temperature on CAT activity (**A**) and stability (**B**). Effect of pH on CAT activity (**C**) and stability (**D**). (**A**) CAT assay was conducted at different temperatures (4–60 °C) at a pH of 6.5 for 20 min. The temperature that gave the maximal CAT activity was set as 100%. (**B**) CAT activity was determined at a pH of 6.5 at 37 °C for 20 min after being incubated at different temperatures (4–90 °C) for 1 h. The activity without incubation was set to 100%. (**C**) CAT assay was conducted at different pH values (1.0–9.0) and at 37 °C for 20 min. The pH values that gave the maximal CAT activity was set as 100%. (**D**) Enzyme activity was determined at a pH of 6.5 and at 37 °C for 20 min after being incubated at different pH values (1.0–9.0) for 6 h. The activity without incubation was set to 100%. The resulting values represent the mean values of two independent experiments.

The CATs from *Cy. rhodanensis* exhibited distinctive differences in optimal temperature and thermostability among strains. The CAT of *Cy. rhodanensis* A22.3 showed the highest activity at 50 °C, which is a higher optimal temperature than that for the CAT obtained from *Cy. rhodanensis* A45.3; it also showed a broader range of temperature optima. The CAT of *Candida* sp. A39.3 had the same optimal temperature at 30 °C as that of *Cy. rhodanensis* A45.3, whereas the CAT from *D. hansenii* A45.1 had an optimal temperature of 37 °C (Figure 3A).

In agreement with the optimal temperature results, the CAT from *Cy. rhodanensis* A22.3 showed the greatest half-life thermostability at 70 °C, whereas that from *Cy. rhodenensis* A45.3 exhibited a lower thermostability, with a half-life at 60 °C. The half-life thermostabilities of CATs from *Candida* sp. A39.3 and *D. hansenii* were in the same range and were the lowest ones, with values of approximately 50 °C. Notably, CAT obtained from *Cy. rhodanensis* A22.3 retained its activity at above 80 °C, whereas others were completely inactive at these temperatures (Figure 3B).

The CATs from *Cy. rhodanensis* A22.3 and *Candida* sp. A39.3 revealed distinctive profiles of pH optimum, whereas that from *Cy. rhodanensis* A45.3 showed a profile similar to that of *D. hansenii* A45.1. It is remarkable that *Cy. rhodanensis* A22.3 produced the CAT that was active at acidic conditions, with an optimum pH value at 2.0; more than 80% relative activity was detected at pH 2.5 and pH 3.0, respectively. On the other hand, the CAT from *Candida* sp. A39.3 preferred neutral to alkaline conditions, as its pH optimum was found at pH 7.0, with more than 80% relative activity at pH values up to 9.0. Comparison of the pH optima between the CATs from *D. hansenii* A45.1 and *Cy. rhodanensis* A45.3 revealed that both had similar pH optima, ranging from pH 5.0–6.0, but the CAT from *Cy. rhodanensis* A45.3 showed a broader range of pH optima as it was still active, with more than 80% relative activity, at pH values ranging from 5.0–9.0 (Figure 3C).

Not only the pH optimum but also the pH stability results revealed that the CAT from *Cy. rhodanensis* A22.3 was active and stable under acidic conditions. The enzyme was stable and retained its original activity of above 80% at a pH range of 2.0 to 4.0 under 37 °C for 6 h. In contrast, the CATs from *Candida* sp. A39.3, *D. hansenii* A45.1, and *Cy. rhodanensis* A45.3 showed a similar profile of pH stability, with stability between pH 4.0 and 9.0. Remarkably, the CAT from *D. hansenii* A45.1 was not stable under pH values below pH 4.0 (Figure 3D).

### 3.4. Effects of Cations on CAT Activity

Most CATs were not affected or partially inhibited by the tested monovalent (Na^+^ and K^+^) and some divalent cations (Ca^2+^, Mg^2+^ and Zn^2+^). Divalent cations, including Mn^2+^, Co^2+^, and Ni^2+^, partly enhanced the activities of all CATs. Among the tested cations, Fe^3+^ was the only trivalent cation that significantly promoted the activities of all CATs, with the highest relative activities ranging from 141.6 ± 0.0 to 170.7 ± 3.2%. On the other hand, Cu^2+^ was partially inhibited all CATs by approximately 20–40% compared to the trials without supplementation. Particularly, Al^3+^ exhibited an inhibitory effect toward all CATs, since the enzymes retained approximately 20–50% relative activity (Table 1).

### 3.5. Effects of Organic Solvents on Enzyme Stability

All CATs were not stable when incubated in all tested organic solvents, specifically acetone; however, the residual activities toward each organic solvent differed depending on the CAT-producing yeast strains. The CATs from *Cy. rhodanensis* tended to be more stable than those from *Candida* sp. A39.3 and *D. hansenii* A45.1. They retained more than 50% of the original activity after incubation in all tested alcohols. The CAT from *Candida* sp. A39.3 was more stable in propanol and butanol than that of *D. hansenii* A45.1 (Table 2).

### 3.6. Substrate Specificity and Biotransformation of Catechin Derivatives

**Figure 4:**
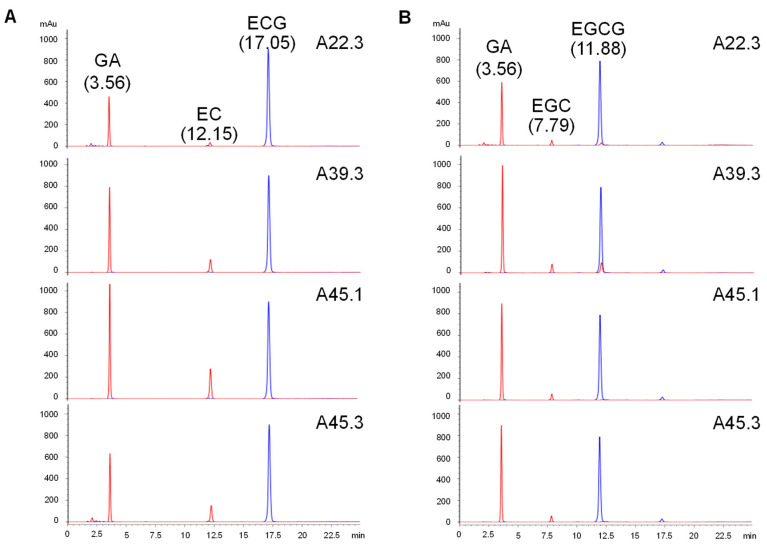
HPLC chromatograms of the biotransformation of ECG (**A**) and EGCG (**B**) by different CATs. Blue line represents substrate blank; Red line represents enzyme-substrate reaction.

In addition to methyl gallate and propyl gallate, two additional substrates were used to determine the substrate specificity of each CAT. Except for CAT from *Cy. rhodanensis* A22.3, all CATs showed a lower activity when reacting with propyl gallate. Interestingly, all CATs had a higher specificity toward ECG and EGCG than methyl gallate. Furthermore, all CATs showed the highest substrate specificity with ECG (Table 3). The highest relative activities toward ECG were 141.0 ± 2.8, 185.6 ± 8.4, 290.3 ± 0.3, and 201.6 ± 2.8% relative activity for *Cy. rhodanensis* A22.3, *Candida* sp. A39.3, *D. hansenii* A45.1, and *Cy. rhodanensis* A45.3, respectively. Although the CAT activity was calculated based on gallic acid production of the certain catechin substrates, the HPLC results revealed that all CATs reacted with EGCG to generate EGC and gallic acid (GA) in addition to EC and GA from ECG (Figure 4). A small amount of the initial substrates was detected after the end of the incubation period.

### 3.7. Repeatability of CATs

**Figure 5:**
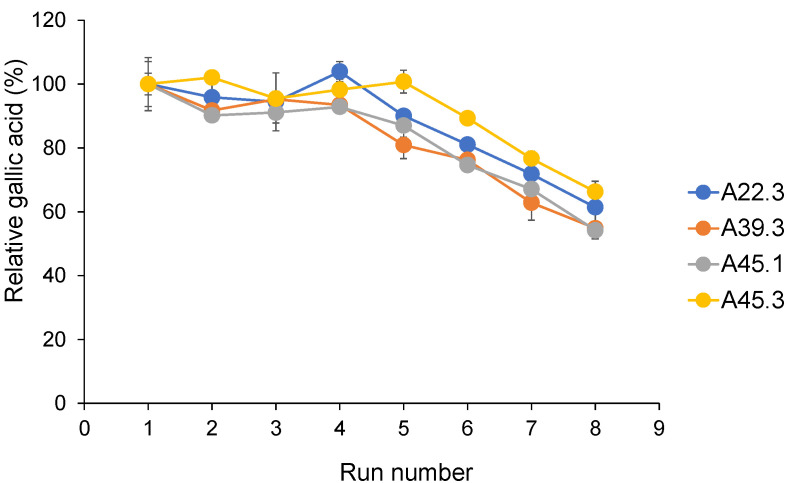
Operational stability of CATs under repeated use. Each CAT was determined using standard assay conditions with an incubation time of 20 min in a repeated batch process. After that, it was recovered by centrifugation and subsequently used for another batch operation. The initial concentration of gallic acid obtained from the first batch was set to 100%. The resulting values represent the mean values of two independent experiments.

All CATs retained almost 100% relative gallic acid after five runs of gallic acid production from methyl gallate. The relative gallic acid values of all CATs were decreased to 50% after the CATs were reused for eight runs (Figure 5).

## 4. Discussion

A total of nine tannin-tolerant and tannase-producing yeasts were isolated from Miang, traditionally fermented tea leaves from northern Thailand. They were two strains of *Cy. rhodanensis* (strains A22.3, A45.3), five strains of *D. hansenii* (strains A28.1, A28.2, A42, A45.1 and A46), *Candida* sp. A39.3 and *S. ruineniae* A45.2 [10]. In addition to *S. ruineniae* A45.2, only *D. hansenii* A45.1 was selected as a representative of *D. hansenii* since it was able to grow at 30 °C as the highest growth temperature. Here, the effects of temperature on the growth of four yeast strains were investigated to determine thermotolerant yeasts for further applications, especially probiotic applications, as accomplished in a previous study [18]. Our results show that *Cy. rhodanensis* strains are of particular interest due to their high optimum growth temperature of 39 °C; above this temperature, growth was partially limited. In contrast, *Candida* sp. A39.3 and *D. hansenii* A45.1 showed optimum growth at 30 °C. One of the most important criteria to evaluate the growth temperature of yeasts is a feasible prediction to achieve an inducible enzyme that may be active and/or tolerant toward high temperatures, as described elsewhere [19]. Tannase is an inducible enzyme produced in the presence of tannins [20], and hence, tannic acid was used as the inducer. When growing in tannic acid, 5 g/L tannic acid had no effect on the growth of all yeasts. All yeast strains were able to grow and produce CAT, although the accumulation of gallic acid in their cultures was different, which might be explained by the presence of gallic acid decarboxylase, which generally converts gallic acid to pyrogallol and is active in the presence of oxygen [21]. This result is in accordance with previous reports [7,22]. Yet, it is possible to produce pyrogallol from tannic acid and other form of tannins by CATs from the *Cy. rhodanensis* strains.

In this study, all CATs were used for comparative biochemical characterization since it was previously determined that the properties of CAT acted equally or better than the purified enzyme, particularly in terms of enzyme stability [10]. Differences in enzyme properties were found among and within yeast species. The pH and temperature optima and stabilities of *Cy. rhodanensis* A22.3, as well as the substrate specificity, differed from those of *Cy. rhodanensis* A45.3, whereas other properties, including cation requirement and solvent stability, were similar. Surprisingly, CAT from *Cy. rhodanensis* A22.3 revealed different biochemical properties compared to other CATs in this research study and to the reported yeast tannases [6,9,10,11]. No microbial tannase was found to be active and stable at a pH value of 2.0. Based on the literature, most fungal tannases are generally active at pH values between 4.0 and 6.0, whereas bacterial tannases are typically active at pH values between 4.0 and 7.0 [5,23], with the exception of lactobacilli tannases such as *L. plantarum*, *L. paraplantarum*, and *L. pentosus* tannases. The reported yeast tannases as well as tannases from *Candida* sp. A39.3, *D. hansenii* A45.1, and *Cy. rhodanensis* A45.3 revealed their pH optima with values ranging from 4.5 to 7.0 (Table 4). Therefore, tannase of *Cy. rhodanensis* A22.3 can be classified as the first acid-stable tannase.

The temperature optima and stabilities of the CATs obtained from this study were in the same range as those reported for other microbial tannases. After fungal tannases, yeasts typically produce tannases that are more active and stable at high temperatures than bacterial tannases [5,23,24,25].

Cations act as salt and complex ions to preserve tannase conformation or to stabilize the binding of a specific substratum molecule as a cofactor [1] rather than to promote the catalytic reaction of the enzyme. It is believed that the rigid conformation of the enzyme molecule can be maintained by a salt or ion bridge via a cluster of carboxylic groups of the enzyme. On the other hand, transition and heavy metals generally inhibit most tannases (example, Fe^2+^, Fe^3+^, Cu^2+^, and Hg^2+^), most likely because of the binding to thiol groups or the interaction with tryptophan residues or the carboxy group of amino acids in the enzyme molecule [26,27].

This evidence is partly in agreement with the results of this study since Fe^3+^ significantly enhanced the activity of all CATs, in contrast to Al^3+^, whereas Cu^2+^ partially inhibited all CATs. Similar results have been found for yeast tannases, namely *S. ruineniae* and *R. diobovatum* [10,11]. In a previous study, Cu^2+^ and Fe^3+^ promoted the activity of tannase from *K. marxianus* [9], whereas both showed an inhibitory effect on tannase from *B*. *adeninivorans* [8]. It has been suggested that tryptophan, which is located at the catalytic site of tannase, is highly sensitive to salt interaction and the microenvironment of the enzyme and thus directly affects tannase activity [28].

The protic solvents may facilitate substrate availability of the active site of tannase and enhance enzyme activity at the same time. They can absorb the essential water molecule from the enzyme, which in turn reduces tannase activity [26]. In the transesterification process for the production of gallate esters, the reaction is typically initiated in the presence of non-aqueous solvents to enhance substrate availability [25]. Therefore, solvent-tolerant enzymes are required for certain processes. Here, most tannases were partially inhibited by the tested protic solvents, especially acetone, with the exception of tannases from *Cy. rhodanensis* A45.3. It is suggested that the solvent tolerance of tannase varies depending on the strains and species.

Interestingly, all CATs showed high activity toward tea catechins, including EGCG and, especially, ECG. Generally, tea catechins are composed of the four main components epicatechin (EC), epigallocatechin (EGC), epicatechin gallate (ECG), and epigallocatechin gallate (EGCG). Moreover, they have their epimer, namely catechin (C), gallocatechin (GC), catechin gallate (CG) and gallocatechin gallate (GCG), respectively. All tea catechins show antioxidant activity and bioavailability, especially when they are in epimer forms [29]. The stoichiometry for EGCG degradation by tannase is formation of EGC and gallic acid, whereas EC and gallic acid are two major products formed by the degradation of ECG [30]. In recent decades, only a yeast *R. diobovatum* Q95 has been reported to produce tannase, which is active toward epicatechins [11]; however, its relative activity to catalyze the hydrolyses of EGCG and ECG was lower than that of the tannases reported in this study. The high substrate specificity toward epicatechins can be explained by the source of tannin-tolerant and tannase-producing yeasts, which may be an enrichment material for microorganisms and, at the same time, act as an inducer for tannase production. All CAT-producing yeasts have been isolated from astringent Miang [13] which were produced by fermentation of young tea leaves for up to 4 weeks [14]. The profile of Miang catechins has been described in a previous study. There were dynamic changes in Miang catechin, especially the biotransformation of EGCG to EGC and ECG to EC during the production process [16]. With regard to the substrate specificity results, all CATs could be used for biotransformation [31], antioxidant activity enhancement, and extraction of tea catechins [32], as well as the improvement of the sweet aftertaste of tea products [4]. Regarding the properties of CAT from *Cy. rhodanensis* A22.3, this is the first report on acid-stable tannase from yeast that can be used as a feed additive tannase for the development of feed formulation.

According to the nature of tannases that are produced and linked on the cell wall surface of yeasts, it can be implied that the CATs can be used as an immobilized enzyme, which is insoluble and can easily be separated from the biotransformation process. In addition, the CATs are reusable, suggesting their application in the further extraction and biotransformation of tea catechins as well as in gallic acid and pyrogallol production.

## 5. Conclusions

In conclusion, this study shows the versatile properties of CATs obtained from tannin-tolerant and tannase-producing yeasts. They can be applied in numerous fields such as food, feed, and pharmaceutical industries. However, these CATs have a high potential in the biotransformation and extraction of tea catechins due to their substrate specificity. In addition, all CATs are likely to be stable and can be used as immobilized enzymes, which will benefit production processes in terms of enzyme recovery and recycling.

## Figures and Tables

**Table 1:** Effect of cations on CAT activity.

Cations (5 mM)	Relative Activity (%)			
	*Cy. rhodanensis* A22.3	*Candida* sp. A39.3	*D. hansenii* A45.1	*Cy. rhodanensis* A45.3
Na^+^	91.3 ± 4.3 ^d^	97.9 ± 3.6 ^cd^	105.4 ± 6.9 ^cd^	98.6 ± 6.8 ^d^
K^+^	97.6 ± 4.5 ^cd^	102.1 ± 3.2 ^cd^	96.2 ± 2.3 ^de^	88.9 ± 2.8 ^de^
Ba^2+^	90.5 ± 0.4 ^d^	89.7 ± 4.8 ^d^	84.1 ± 7.7 ^e^	87.8 ± 2.7 ^de^
Ca^2+^	99.5 ± 0.9 c^d^	131.9 ± 2.5 ^b^	100.6 ± 0.1 ^cde^	98.0 ± 5.6 ^d^
Mg^2+^	102.1 ± 2.5 ^cd^	111.3 ± 6.0 ^c^	86.9 ± 1.7 ^de^	80.7 ± 1.4 ^e^
Cu^2+^	70.7 ± 6.0 ^e^	63.4 ± 2.2 ^e^	88.2 ± 1.0 ^de^	60.3 ± 2.4 ^f^
Mn^2+^	118.9 ± 0.8 ^b^	154.1 ± 5.9 ^a^	139.9 ± 8.6 ^b^	171.8 ± 5.3 ^a^
Co^2+^	119.5 ± 1.1 ^b^	137.9 ± 3.1 ^b^	117.7 ± 2.2 ^c^	144.1 ± 0.0 ^b^
Ni^2+^	119.0 ± 0.8 ^b^	111.9 ± 1.1 ^c^	100.5 ± 6.0 ^cde^	128.7 ± 7.2 ^c^
Zn^2+^	107.2 ± 1.5 ^bc^	90.5 ± 1.0 ^d^	94.8 ± 4.7 ^de^	102.0 ± 2.2 ^d^
Al^3+^	53.5 ± 0.2 ^f^	36.8 ± 0.9 ^f^	27.8 ± 4.0 ^f^	18.2 ± 2.0 ^g^
Fe^3+^	146.3 ± 4.7 ^a^	142.5 ± 4.8 ^ab^	170.7 ± 3.2 ^a^	141.6 ± 0.0 ^bc^
Control	100.0 ± 6.8 ^cd^	100.0 ± 0.3 ^cd^	100.0 ± 3.1 ^cde^	100.0 ± 2.1 ^d^

Different lowercase letters within a column indicate significant differences in CAT activity at *p* < 0.05. The resulting values represent the mean values of two independent experiments.

**Table 2:** Effects of different organic solvents on CAT stability.

Solvents	Residual Activity (%)			
	*Cy. rhodanensis* A22.3	*Candida* sp. A39.3	*D. hansenii* A45.1	*Cy. rhodanensis* A45.3
Methanol	64.4 ± 1.3 ^b^	64.9 ± 5.6 ^bc^	69.4 ± 2.3 ^b^	90.5 ± 3.3 ^b^
Ethanol	65.4 ± 3.9 ^b^	55.7 ± 1.9 ^bc^	55.4 ± 3.0 ^c^	79.2 ± 1.7 ^c^
Propanol	49.3 ± 2.0 ^c^	34.4 ± 2.5 ^d^	13.4 ± 1.9 ^e^	52.1 ± 1.8 ^d^
Butanol	62.8 ± 1.0 ^b^	47.8 ± 4.4 ^cd^	25.2 ± 3.2 ^d^	74.4 ± 2.3 ^c^
Acetone	47.0 ± 4.0 ^c^	17.4 ± 1.9 ^d^	10.9 ± 1.4 ^e^	32.8 ± 1.5 ^e^
Control	100.0 ± 1.1 ^a^	100.0 ± 1.5 ^a^	100.0 ± 0.7 ^a^	100.0 ± 1.3 ^a^

Different lowercase letters within a column indicate significant differences in residual CAT activity at *p* < 0.05. The resulting values represent the mean values of two independent experiments.

**Table 3:** Substrate specificity.

Strains	Relative Activity (%)			
	Methyl Gallate	Propyl Gallate	EGCG	ECG
*Cy. rhodanensis* A22.3	100.0 ± 3.1 ^b^	95.5 ± 4.3 ^b^	139.2 ± 3.1 ^a^	141.0 ± 2.8 ^a^
*Candida* sp. A39.3	100.0 ± 5.2 ^c^	75.7 ± 5.5 ^d^	160.3 ± 2.3 ^b^	185.6 ± 8.4 ^a^
*D. hansenii* A45.1	100.0 ± 5.5 ^c^	73.6 ± 7.8 ^d^	149.6 ± 1.0 ^b^	290.3 ± 0.3 ^a^
*Cy. rhodanensis* A45.3	100.0 ± 4.1 ^c^	75.4 ± 7.5 ^d^	153.7 ± 5.5 ^b^	201.6 ± 2.8 ^a^

Different lowercase letters within a row indicate significant differences in relative CAT activity at *p* < 0.05. The resulting values represent the mean values of two independent experiments.

**Table 4:** Summary comparison of CAT properties with the reported yeast tannases.

Microorganisms	Optimal pH	Optimal Temperature	pH Stability	(t_1/2_) Thermostability	References
*Candida* sp.	6.0	50 °C	4.0–8.0 at 13 °C, 16 h	60 °C, 10 min	[6]
*B. adeninivorans*	7.0	40 °C	5.0–7.0, 10 min	Stable at 30–40 °C, 10 min	[8]
*K. marxianus*	4–4.5	35 °C	4–5.5, at 30 °C, 30 min	70 °C, 1 h	[9]
*S. ruineniae* A45.2	7.0	40 °C	5.0–9.0 at 37 °C, 6 h	75 °C, 1 h	[10]
*R. diobovatum*	4.5	40 °C	4.5–5.5, at 40 °C, 12 h	55–60 °C, 12 h	[11]
*Cy. rhodanensis* A22.3	2.0	45 °C	2.0–4.0, at 37 °C, 6 h	70 °C, 1 h	This study
*Cy. rhodanensis* A45.3	6.0	20–40 °C	4.0–9.0, at 37 °C, 6 h	60 °C, 1 h	This study
*Candida* sp. A39.3	7.0	20–30 °C	5.0–9.0, at 37 °C, 6 h	50 °C, 1 h	This study
*D. hansenii* A45.1	5.0	20–40 °C	4.0–9.0, at 37 °C, 6 h	50 °C, 1 h	This study

## Data Availability

Not applicable.
